# Sub-Exome Target Sequencing in a Family With Syndactyly Type IV Due to a Novel Partial Duplication of the *LMBR1* Gene: First Case Report in Fujian Province of China

**DOI:** 10.3389/fgene.2020.00130

**Published:** 2020-02-28

**Authors:** Lijing Shi, Hui Huang, Qiuxia Jiang, Rongsen Huang, Wanyu Fu, Liangwei Mao, Xiaoming Wei, Huanhuan Cui, Keke Lin, Licheng Cai, You Yang, Yuanbai Wang, Jing Wu

**Affiliations:** ^1^Department of Ultrasound, Quanzhou Women’s and Children’s Hospital, Quanzhou, China; ^2^BGI Genomics, BGI-Shenzhen, Shenzhen, China; ^3^Prenatal Diagnosis Center, Quanzhou Women’s and Children’s Hospital, Quanzhou, China; ^4^BGI-Wuhan Clinical Laboratories, BGI-Shenzhen, Wuhan, China; ^5^State Key Laboratory of Biocatalysis and Enzyme Engineering, College of Life Sciences, Hubei University, Wuhan, China; ^6^BGI-Guangzhou Medical Laboratory, BGI-Shenzhen, Guangzhou, China

**Keywords:** syndactyly type IV, *LMBR1*, triphalangeal thumb-polysyndactyly syndrome, tibial and fibulal shortening, sub-exome target sequencing

## Abstract

Syndactyly is one of the most frequent hereditary limb malformations with clinical and genetical complexity. Autosomal dominant syndactyly type IV (SD4) is a rare form of syndactyly, caused by heterozygous mutations in a sonic hedgehog (*SHH*) regulatory element (*ZRS*) which resides in intron 5 of the *LMBR1* gene on chromosome 7q36.3. SD4 is characterized by complete cutaneous syndactyly of the fingers, accompanied by cup-shaped hands due to flexion of the fingers and polydactyly. Here, for the first time, we reported a large Chinese family from Fujian province, manifesting cup-shaped hands consistent with SD4 and intrafamilial heterogeneity in clinical phenotype of tibial and fibulal shortening, triphalangeal thumb-polysyndactyly syndrome (TPTPS). We identified a novel duplication of ∼222 kb covering exons 2–17 of the *LMBR1* gene in this family by sub-exome target sequencing. This case expands our new clinical understanding of SD4 phenotype and again confirms the feasibility to detect copy number variation by sub-exome target sequencing.

## Background

Syndactyly is one of the most frequent hereditary limb malformations (Tonkin, 2009). The occurrence rate of syndactyly is variable due to geographical and registry differences, ranging from 1.1/10,000 in the northern Netherlands (Vasluian et al., 2013) to 1.3/10,000 in New York State (Goldfarb et al., 2017). The incidence of syndactyly in China is 7.4/10,000 in 1998–2009 (Sun et al., 2011). Currently, five pathogenic genes for syndactyly have been reported, which are associated with nonsyndromic syndactyly types I-c, II-a, II-b, III, IV, V, VII, VIII (Ahmed et al., 2017). Syndactyly type IV (SD4; OMIM 186200), also known as Haas polysyndactyly, is an autosomal dominant condition occurring as a result of heterozygous mutations of a sonic hedgehog (*SHH*) regulatory element, designated *ZRS*, within intron 5 of the *LMBR1* gene (limb development membrane protein 1 gene) on chromosome 7q36.3 (Lettice et al., 2003). SD4 is characterized by complete cutaneous syndactyly of all fingers, cup-shaped hands due to flexion of the fingers, accompanied by polydactyly. The phenotypic and the genotypic variability in syndactyly may pose a diagnostic challenge for clinicians. Point mutations within the *ZRS* region have been described associated with preaxial polydactyly or triphalangeal thumb with preaxial polydactyly (Farooq et al., 2010; Albuisson et al., 2011). A large study of Chinese families showed the occurrence of small duplications that affect *ZRS* locus both in SD4 and the more severe triphalangeal thumb-polysyndactyly syndrome (TPTPS) (Klopocki et al., 2008; Wieczorek et al., 2010). Some small duplications (<80 kb) are associated with a more severe phenotype, named Laurin-Sandrow syndrome (Lohan et al., 2014). The *ZRS* which maps approximately 1 Mb away from *SHH* (Lettice et al., 2003) controls the expression of *SHH* in the developing limb and is conserved among mammals and fish (Lettice et al., 2003). The *LMBR1* mutant mouse displayed syndactyly involving digits II to V (Al-Qattan et al., 2013), which is in line with the phenotypes of SD4.

Until now, 14 cases, mainly diagnosed as TPTPS or SD4 or TPTPS accompanying SD4, were reported to have duplication covering *ZRS* locus by different technologies (Dai et al., 2013; Lohan et al., 2014; Liu et al., 2017) (**Supplementary Table 1**). No lower limb dysplasia was reported in these patients, only two SD4 cases were with fibula dysplasia, but the evidences were insufficient: either lack of X-ray image of fibula dysplasia (Wu et al., 2009) or without precise description of the genomic duplication (Sato et al., 2007). Here, we report a large Chinese family with a novel duplication of ∼222 kb covering exons 2–17 of the *LMBR1* gene, underlying that cup-shaped hands were correlated with SD4 and intrafamilial heterogeneity in clinical phenotype by sub-exome target sequencing and imaging evidence.

## Case Presentation

**Figure 1:**
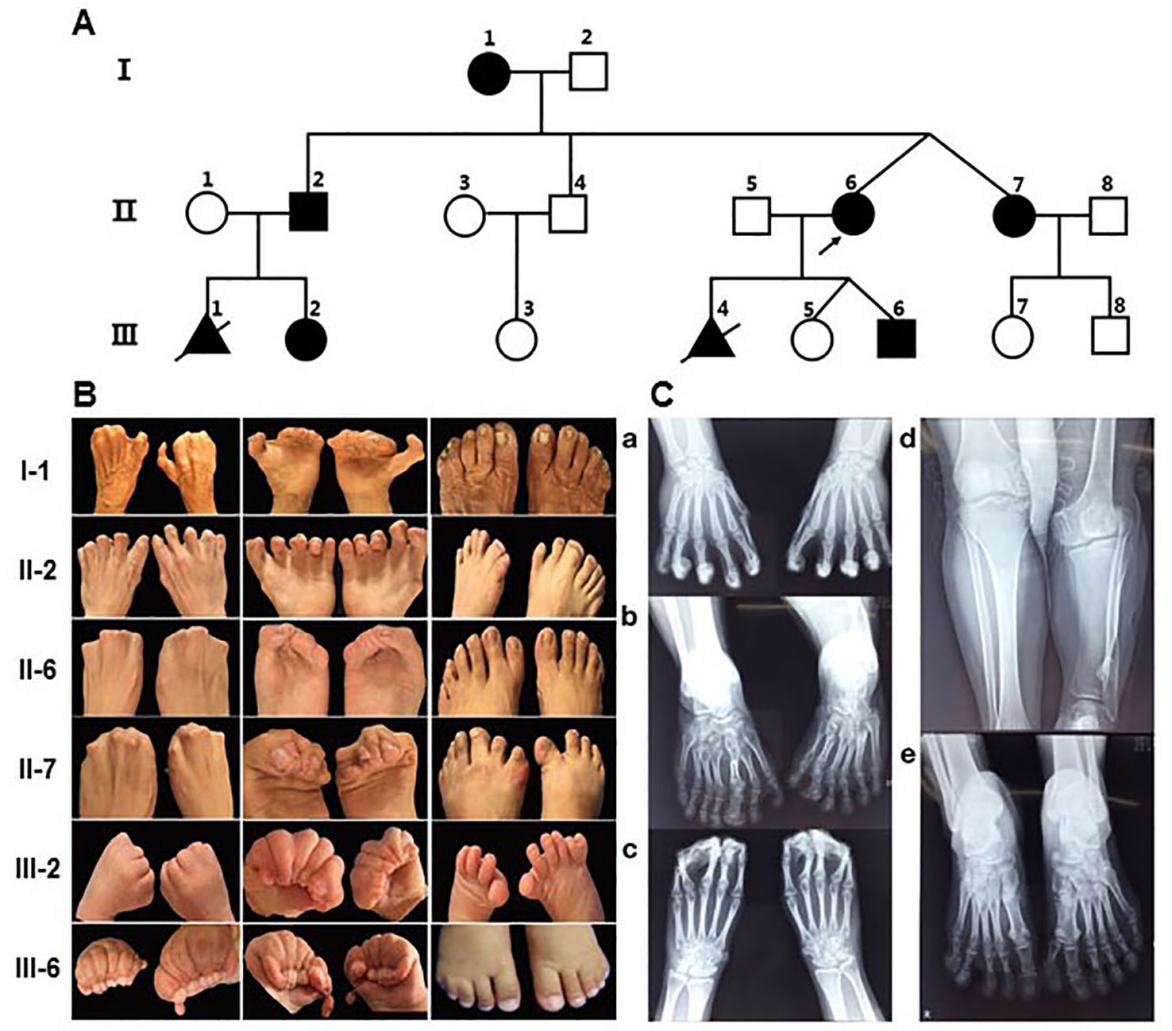
Pedigree of the family with syndactyly and limb features of patients. **(A)** Pedigree of the three-generation family with syndactyly. **(B)** Images of limb features in the family. **(C)** X- ray images of hands and feet PA in II-2 and the proband (II-6). a) Hands PA of II2, postoperation: An extra metacarpal and two phalanges are observed at the lateral part of the first metacarpal on the left and right hand, and the soft tissue of the extra metacarpal and that of thumb is fused. The two hands of the distal phalangeal flexion overlap. b) Feet PA of II2: Both feet are varus, and the tarsal bones are markedly shortened. Extra tarsal and phalanx bones are found on feet. d) Bilateral tibial and fibular PA and LAT of II2: The left side of the tibia is markedly shortened; there is a bony eminence in the local cortex. c) Hands PA of II-6: The middle and the distal phalanx overlap, and the soft tissue of the distal part of both hands is fused. e) Feet PA of II-6: A polydactylism is observed at the lateral part of the hallux on left foot, which contains two sections of short phalange with the formation of the joint between phalanges, proximal phalanx bones, and the first tarsal joint; the size and morphology of the first tarsal bone is normal. The distal phalanx of the fifth toe on left foot enlarges and deforms.

The proband (II-6) (**Figure 1A**) was a 26-year-old woman with twin pregnancy, presented malformed hands and fingers. Both hands have complete syndactyly. The left foot had six toes and a preaxial polydactyly; on the right foot, syndactyly was between the fourth and fifth toe. She had a first pregnancy with a fetus interrupted due to hand malformations identified at ultrasound screening in Quanzhou Woman’s and Children’s Hospital. In a second pregnancy, prenatal ultrasound at 22 weeks revealed two twins both with fetal hands in a fist-shaped posture and the absence of finger-to-finger interval, hands and feet deformity could not be ascertained. Investigating her family history, we found she belongs to a large Chinese family segregating autosomal dominant non-syndromic syndactyly. Eight members from three generations were affected, including three males and five females (**Figure 1A**). Two of these cases (III-1, III-4) had syndactyly confirmed by prenatal ultrasonography and were interrupted (**Figure 1A**). None of the affected family members exhibited intellectual anomalies.

By physical examination, we found all patients had the typical clinical phenotype of SD4: cup-shaped hands caused by the fingers together with cutaneous syndactyly, accompanied by limited flexion of the fingers, but no fusion of phalanges and metacarpus. The clinical phenotypic diversity of our family members is characterized by varying degrees of syndactyly and polydactyly in different fingers, skin fusion, preaxial polydactyly and fused fingernails. In most of the affected patients, the presentation was bilateral and symmetrical, accompanied by syndactyly and polydactyly (**Figure 1B**). Phenotypic spectrum in all affected subjects can be seen in **Supplementary Table 2**. Some affected individuals have other abnormalities in their fingers and lower limb. In appearance, I-1 presented with triphalangeal thumb on her right hand, and the phenotype of II-2 is the most variable. His hands had a complete syndactyly (surgically treated) and triphalangeal thumb, with seven-toes deformity and the asymmetrical syndactyly on the left and right toes and accompanied by 1–2 small toes of both feet, the third big toe shaped like a thumb, appeared limply walking. In addition, III-6 had a congenital cleft lip and palate, which has been treated surgically. Meanwhile, special attention should be paid to differences in the malformations of the twins. The right toe of II-7 is normal and the left toe of II-7 is an extra postaxial toe. The right fourth and fifth toes of II-6 are syndactyly, and the left toe of II-6 are two extra postaxial toes.

Clinical X-ray examination of the proband (II-6) (**Figure 1C-c, e**) and II-2 (**Figure 1C-a, b, d**) revealed the characteristics of the deformity of both hands and feet and the changes of the bilateral tibial and fibular. II-2 is represented by unilateral shortened tibia and fibula (**Figure 1C-d**), triphalangeal thumb on his right hand (**Figure 1C-c**), but it is not sure on his left hand because of all bent and overlapping fingers by X-ray. X-rays from other family members were not available.

## Laboratory Investigations and Test Results

Our research was approved by the institutional review board of Quanzhou Woman’s and Children’s Hospital. Written consent for reporting clinical results was obtained from all the participants. Targeted gene testing of peripheral blood samples from six patients (II-6, III-6, II-7, III-7, II-4, II-2) were performed to make molecular pathogenic diagnosis, genomic DNA were sequenced using a BGISEQ-500 sequencer, which average coverage depth is nearly 400×. The targeted sequences were captured using the Genetic Disease Chip from internal laboratory (named sub-exome target sequencing), which contained 3,299 genes and covered seven genes [*HOXD13*, *FBLN1, ZRS(LMBR1), LRP4, FGF16, BHLHA9, GJA1*] related to syndactyly according to OMIM. The point mutations and copy number variations of genetic diseases-related genes can be detected more comprehensively by the sub-exome target sequencing. The library was prepared by shearing 1 μg of genomic DNA into a small fragment of 200–300 bps of DNA. The methods used for target capture, enrichments, and elution followed previously described protocols with slight modifications. Sequencing was performed using the BGISEQ-500 platform. Bioinformatic analysis included low-quality read removal with SOAPnuke, alignment to UCSC hg19 with BWA, and variant detection with GATK, and variant annotation was conducted as described previously (Liu et al., 2015; Patch et al., 2018; Zheng et al., 2018). This research uses CNVkit software to detect copy number variation (Talevich et al., 2016). This software uses both the on-target reads and the nonspecifically captured off-target reads to calculate log2 copy ratios across the genome for each sample based on controls (**Supplementary Table 3**). We found a heterozygous duplication spanning exons 2–17 of the *LMBR1*gene (NM_022458.3) in four patients of our family (II-6, III-6, II-7, II-2); the duplication was absent from two unaffected members (II-4, III-7). Demonstration of duplication mutation covering exons 2–17 of the *LMBR1* gene analyzed by Integrative Genomics Viewer (IGV) can be seen in **Supplementary Figure 1**. In order to further clarify the breakpoints of duplication and check the probability of larger fragment duplication, proband (II-6) was tested by low-coverage pair-end whole-genome sequencing as previously reported (Dan et al., 2012) (**Supplementary Figure 2**), and the breakpoint mapping and size of duplication mutation (chr17:156,460,343-156,682,575,222Kb; hg19) were confirmed in II-6. This duplicated region covered only exons 2–17 of one OMIM pathogenic gene *LMBR1*.The identified variant was confirmed by quantitative PCR (qPCR) (**Supplementary Table 4**). A part of the protein-coding region (CDS5, CDS8, CDS15) of the *LMBR1* gene was representatively selected for qPCR verification. qPCR for pedigree analysis confirmed the mutation is cosegregation in this family (**Supplementary Figure 3**). According to the inclusion criteria set by the American College of Medical Genetics and Genomics (ACMG) and the Clinical Genome Resource (ClinGen), the duplication of ∼222 kb (chr7:156,460,343-156,682,575) is rated as “pathogenic” by scoring 1A+0; 2A+1; 4H+0.45 and 5D+0.45 (total score: 1.9) (Sun et al., 2008; Dai et al., 2013; Riggs et al., 2019).

## Discussion and Concluding Remarks

### Phenotype and Mechanism

**Figure 2:**
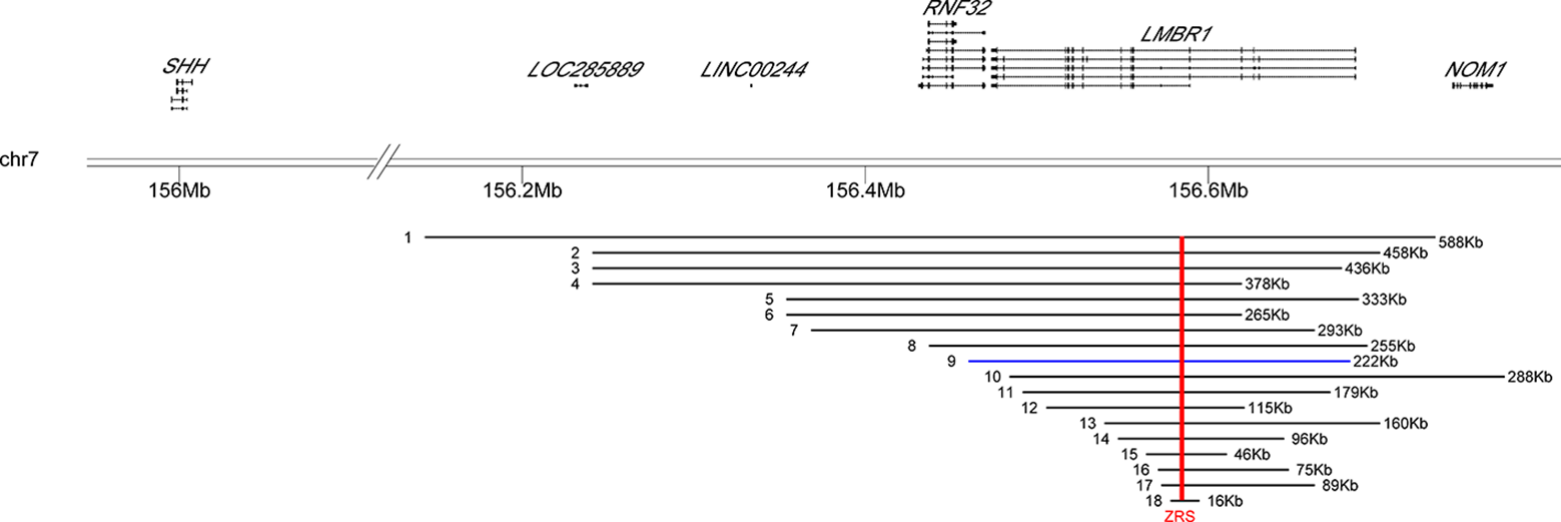
Location distribution of reported duplication variants of *LMBR1* gene in patients. The genomic positions of two genes (*SHH, LMBR1*) were shown above the black horizontal line (representing 7q36.3). The distributions of 17 known duplication variations and the one new found in the study were listed below. Arabic numerals indicated the number of the reported cases in **Supplementary Table 1**. Blue bars represented our case. Red vertical line indicated the position of the *ZRS* in all different duplication regions.

In this study, we found a novel microduplication encompassing exons 2–17 in *LMBR1* gene (covering the SHH limb enhancer *ZRS*) in a large family. The patients showed clinical heterogeneity: most of them manifested SD4 and TPTPS, one of them had severe phenotype with shortened tibia and fibula. Considering previous reports together, it seems that there is no positive correlation between severity of disease and size of duplicating regions (**Figure 2**). For example, TPTPS was observed for the patient with known largest duplication covering *ZRS* (up to 589 kb) while severer Laurin-Sandrow syndrome was found in patient harboring *ZRS* duplication with size less than 80 kb (**Supplementary Table 1**). Thus, we suggest that other genetic or nongenetic determinants might influence the phenotype beyond the duplication. The mechanism how point variants within *ZRS* influence the expression pattern of *SHH* has been well investigated, but the mechanism of duplication variants of the *ZRS* related with phenotype is unclear. There are several speculations for the pathogenesis of duplicated variant. For example, tandem duplications of the *ZRS* might simultaneously increase the copy number of cis-regulatory elements changing the *SHH* expression profile. Meanwhile, duplications might affect 3D structure of the DNA and therefore influence *SHH* expression or affect the interacting between *ZRS* and *SHH* (Spielmann and Mundlos, 2013). As reported previously, limb-specific *SHH* expression was regulated by the *ZRS* enhancer, demonstrated by a topologically associating domain (TAD) over the *SHH*/*ZRS* genomic region *SHH* identified by chromosome conformation capture (5C). It was suggested that close *SHH* and *ZRS* proximity instigate the expression of *SHH* (Williamson et al., 2016). Therefore, the duplications in this locus likely alter the structure of TAD influencing *SHH* expression profile and finally associated with disease, yet more experimental studies are needed for this hypothesis.

One more point to consider is that the reported duplication variations (including our case) were identified by Array CGH, qPCR assays, and targeted sequencing. Although duplicates were found, it was impossible to determine the location of the duplicated regions, or whether they have been inserted into or translocated to other chromosomes, thereby influencing the spatial expression pattern or dosage of SHH during limb bud development. Future applications of transcriptome sequencing or whole-genome sequencing may help answer this question.

### Treatment and Management of Syndactyly

The treatment and management of syndactyly is still by surgical correction. Because of varying degrees of deformity and functional requirements by different patients, it must take into account effects on psychological, economic, and social aspects of the patients, and a higher incidence of complications was observed in patients undergoing complex syndactyly repair (McQuillan et al., 2017); one reported patient with SD4 had 18 surgical corrections (Wieczorek et al., 2010). Therefore, the best prevention for family members with syndactyly is popular science education of genetic diseases and genetic counseling from professionals. Prenatal tests may be offered to at-risk couples.

### A Potential Application in Diagnosis of Genetic Diseases

Reported testing methods for duplication variation were array CGH, qPCR assays, or linkage and haplotype analysis (Sun et al., 2008; Wu et al., 2009), we first used sub-exome target sequencing (average coverage depth nearly 400×) to test both copy number variant (CNV) and single nucleotide variant (SNV) mutations. The duplication segments of exons are given relatively accurately, although discontinuous exon duplication was occasionally found. Undeniably, that is the disadvantage of target capture sequencing due to capture efficiency issues. But the reliability of the results was again confirmed by low-coverage pair-end whole-genome sequencing and qPCR. This work demonstrates that sub-exome target sequencing could be an alternative application in the diagnosis of genetic diseases. Meanwhile, this method also needs mutual verification by other methods in order to be used as a candidate for clinical applications.

In summary, we first described an affected family with heterozygous duplication of exons 2–17 in *LMBR1* gene by sub-exome capture sequencing, who presented with SD4 accompanied with a diverse phenotype of triphalangeal thumb, and tibia and fibula shortening in Quanzhou City, Fujian Province. It again confirmed the genetic homogeneity of TPTPS and SD4 and demonstrated intrafamilial phenotypic heterogeneity.

## Data Availability Statement

The datasets used and/or analyzed during the current study are available from the corresponding authors on reasonable request. The data reported in this study are available in the CNGB Nucleotide Sequence Archive (CNSA: https://db.cngb.org/cnsa; accession number CNP 0000157).

## Ethics Statement

Our research was approved by the institutional review board of Quanzhou Woman’s and Children’s Hospital. Written consent for reporting clinical results was obtained from all the participants.

## Author Contributions

LM, HC, and XW conducted the next generation sequencing, NGS procedure and subsequent analysis/interpretation. QJ and WF conducted the genetic counseling process and the follow-up. RH and WF contributed to physical examination and clinical X-ray examination. KL performed image modification. LC and YY conducted the qPCR analysis. YW and HH instructed and supervised this study. The manuscript was drafted by LS and edited by JW. All authors have read and approved the manuscript.

## Conflict of Interest

Author JW and HH were employed by company BGI Genomics, BGI-Shenzhen.

The remaining authors declare that the research was conducted in the absence of any commercial or financial relationships that could be construed as a potential conflict of interest.

## Supplementary Material

Supplementary Figure 1 Demonstration of duplication covering exons 2-17 of LMBR1 gene tested by low-coverage pair-end whole-genome sequencing and sub-exome target sequencing.

Supplementary Figure 2 The breakpoint mapping of duplication mutation on chromosome 7q36.3.

Supplementary Figure 3 Quantitative real-time PCR validation of partial LMBR1 duplication.
