# Supplementary figures and images for: Sub-Exome Target Sequencing in a Family With Syndactyly Type IV Due to a Novel Partial Duplication of the LMBR1 Gene: First Case Report in Fujian Province of China
Source: Front Genet. 2020 Feb 28;11:130. doi: 10.3389/fgene.2020.00130 (PMC7058806; doi:10.3389/fgene.2020.00130)

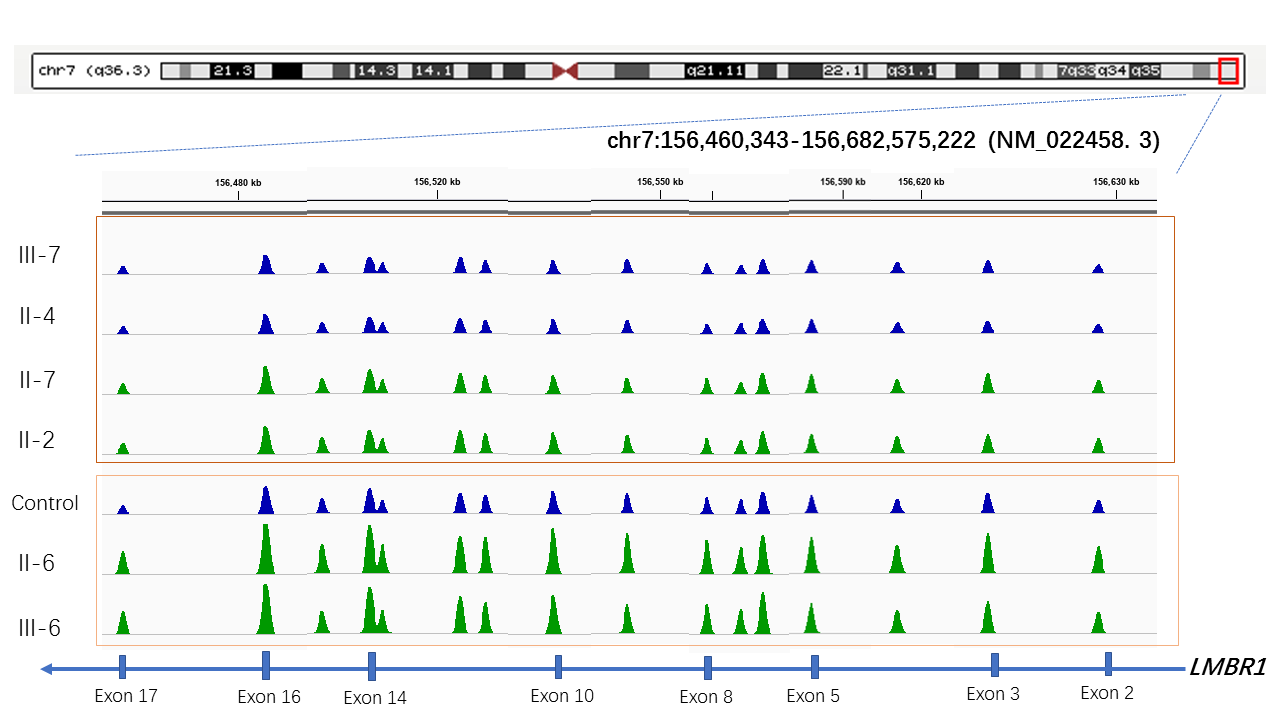

Supplement: Supplementary Figure 1 — Demonstration of duplication covering exons 2-17 of LMBR1 gene tested by low-coverage pair-end whole-genome sequencing and sub-exome target sequencing. [file Image_1.tif]

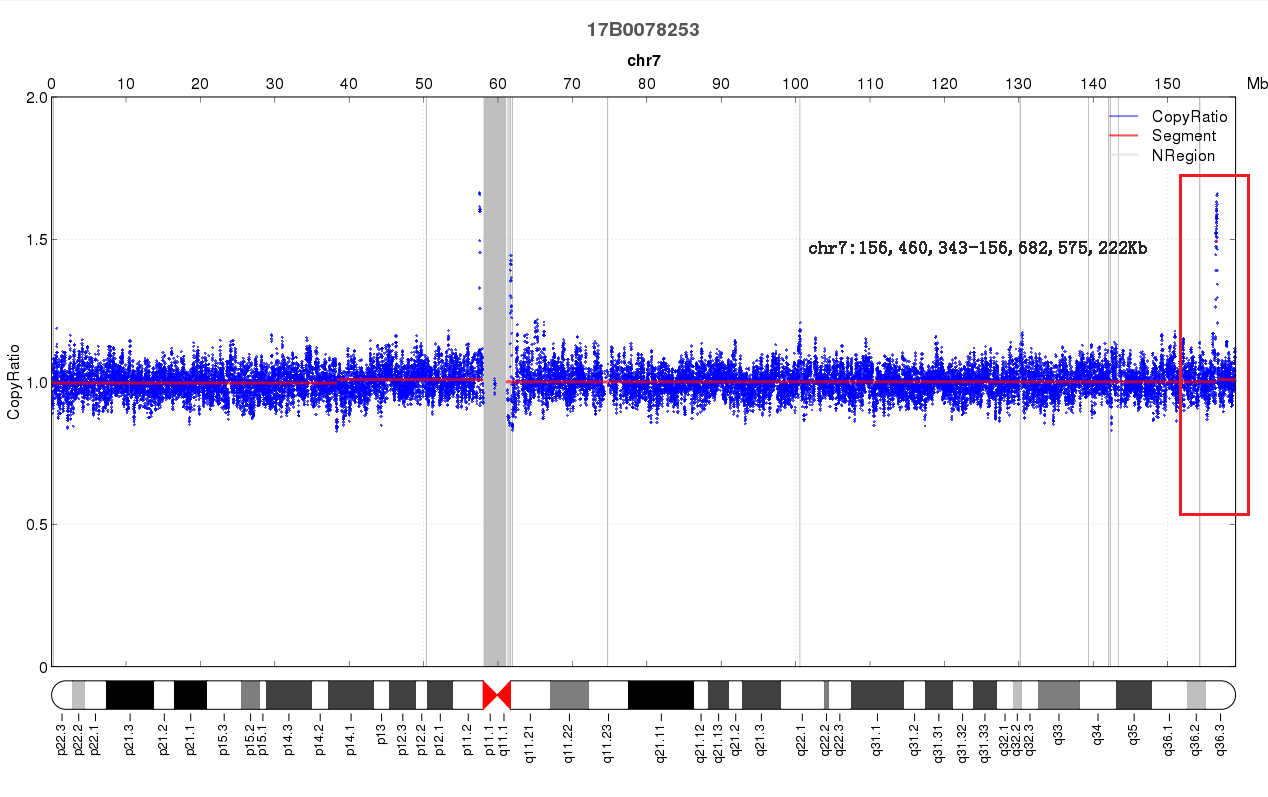

Supplement: Supplementary Figure 2 — The breakpoint mapping of duplication mutation on chromosome 7q36.3. [file Image_2.tif]

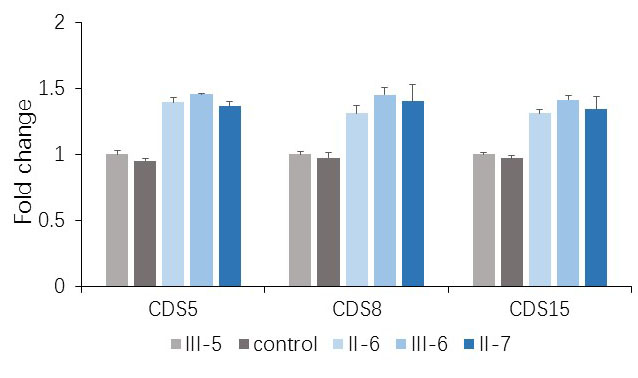

Supplement: Supplementary Figure 3 — Quantitative real-time PCR validation of partial LMBR1 duplication. [file Image_3.jpeg]
